# Biomarkers defining probability of receiving second-line targeted therapy in metastatic renal cell carcinoma

**DOI:** 10.1007/s12032-018-1148-x

**Published:** 2018-05-08

**Authors:** Pawel Chrom, Maciej Kawecki, Rafal Stec, Lubomir Bodnar, Cezary Szczylik, Anna M. Czarnecka

**Affiliations:** 1 Department of Oncology, Military Institute of Medicine, Szaserow 128, 04-141 Warsaw, Poland; 2 Present Address: Maria Sklodowska-Curie Memorial Cancer Center and Institute of Oncology, Wawelska 15, 00-001 Warsaw, Poland; 3 Present Address: Medical University of Warsaw, Zwirki i Wigury 61, 02-091 Warsaw, Poland

**Keywords:** Metastatic renal cell carcinoma, Probability calculator, Second-line, Sequential treatment, Tyrosine kinase inhibitor

## Abstract

In order to facilitate long-term treatment decisions, we aimed to define biomarkers defining the probability of receiving second-line (SL) targeted therapy (TT) in patients with metastatic renal cell carcinoma (mRCC) based on their characteristics present at first-line TT initiation. We analysed 152 consecutive mRCC patients treated and used multivariable binominal logistic regression to identify factors contributing to the probability of receiving SL TT. Final model was assessed with bias-corrected indices (Nagelkerke’s *R*^2^ and area under receiver operating characteristic curve [AUC]) and two bootstrap procedures were used for internal validation. Factors associated with the probability of SL TT eligibility were the presence of brain metastases (odds ratio [OR] 0.084, 95% confidence interval [CI] 0.010–0.707), number of metastatic sites (OR 0.740, 95% CI 0.575–0.953 per each site), platelet count (OR 0.971, 95% CI 0.947–0.997, per 10^4^/ml), lactate dehydrogenase level (OR 0.952, 95% CI 0.910–0.997 per 10 units/l), and albumin concentration (OR 1.924, 95% CI 1.057–3.503 per 1 g/dl). We developed on-line calculator that enables practicing clinicians to estimate SL treatment probability (http://www.r-calc.com).

## Introduction

Currently multiple antiangiogenic compounds including bevacizumab (anti- vascular endothelial growth factor [VEGF] antibody), sorafenib, sunitinib and pazopanib (tyrosine kinase inhibitors [TKIs] targeting VEGF receptors) are first-line standard-of-care treatment options of metastatic renal cell carcinoma (mRCC) providing progression-free survival (PFS) benefit as proven in randomised phase III trials [1]. Nevertheless, these antiangiogenic therapies rarely provide complete or long-term responses [2]. About 80% of patients will experience disease progression after first year of treatment, also despite initial partial response (PR) or stable disease (SD) due to the development of treatment acquired resistance [3]. Moreover, 20% of patients present with initial endogenous resistance to TKIs [4, 5]. Out of all patients with mRCC that progress on first-line of treatment, between 20 and 60% will receive second-line therapy [6–10].

National cancer treatment programmes and/or drug prescription registries that cover the whole country populations provided data on new anti-mRCC therapies within real-life patients [8, 9]. Currently, patients who receive second-line therapy are expected to reach median overall survival (OS) over 27 months, while those who are enrolled in three or more lines of treatment may obtain over 43 months of OS and are greatest beneficiaries of RCC targeted therapies (TT). Up to 85% patients are expected to receive sunitinib as first-line treatment [11]. The percentage of patients who receive a second-line treatment is similar between sunitinib (59%) sorafenib (52%) and bevacizumab (79%) [10] treated patients. Majority of patients in SL are treated with everolimus (40–60%) or sorafenib (up to 30%) [11, 12].

Until now, the Memorial Sloan-Kettering Cancer Center (MSKCC) classification score (Motzer Score) and first-line treatment type were considered as only established predictive factors of receiving second-line therapy [10]. Moreover, early progression is also significantly associated with a higher probability of not receiving second-line anti-mRCC treatment [6]. Nevertheless, still little is known on predictive factors of second-line therapy enrolment in RCC patients. Preclinical data suggest that the main downstream effectors of mammalian target of rapamycin signalling cascade—S6RP protein and its phosphorylated form—may become reliable predictive biomarkers of potential response to everolimus [13], but for everyday practice clinical factors seem to be more suited. The goal of our study was to analyse these questions in a series of subsequent RCC patients treated in community-oriented treatment program at institution recognised for strong patient satisfaction scores and standards compliance. We sought to identify pre-treatment clinical parameters that could help predicting the likelihood of a patient receiving second-line therapy and to develop a toll—calculator—enabling patients stratification.

## Materials and methods

### Patients

Consecutive mRCC patients who started treatment with first-line TT between November 2009 and March 2016, in the Department of Oncology, Military Institute of Medicine in Warsaw, Poland were included in the analysis. Patients with any histological RCC subtype with no other primary malignancies and no adjuvant therapy were eligible. Additionally, patients who were treated with interferon-based immunotherapy prior to the initiation of first-line TT were included; however, IFN was not counted as a line of treatment. Patients were assigned to the second-line (SL) group if they had received any of the second-line TT therapy, or to the non-SL group, if they had not received any therapy beyond first-line. Patients with unknown status of second-line TT were excluded from analysis. This group comprised of patients who (1) continued treatment as had not progressed on first-line TT at the time of the final data collection or (2) discontinued treatment due to toxicity/consent withdrawn, not progression or (3) were lost to follow-up before second-line TT initiation. Inclusion criteria for FL and SL covered adequate organ function as described before [14–19].

The individual medical records were analysed. The institutional ethics committee approved the study (agreement no. 48/WIM/2014). Due to retrospective design of the analysis, individual informed consent was not required.

### Outcomes and statistical methods

The status of second-line TT (received versus not received) was a dependent binary variable for the main analysis. The other assessed outcomes were (1) OS which was defined as the time from the initiation of first-line TT to death from any cause, (2) PFS which was defined as the time from the initiation of first-line TT to disease progression according to the Response Evaluation Criteria in Solid Tumours (RECIST), version 1.1, or death from any cause, and (3) post-progression survival (PPS), which was defined as the time from disease progression on first-line TT to death from any cause. Medians and ranges were used to describe continuous variables whereas frequencies and percentages were used to describe categorical variables. The differences in baseline characteristics between the SL and non-SL groups were assessed using the U-Mann-Whitney test for continuous variables and the Pearson Chi-Square or the Fisher’s exact test (in the case of five or less expected frequencies in each cell of a studied contingency table) for categorical variables. Distributions of OS, PFS and PPS were estimated using the Kaplan–Meier product-limit method; their medians with calculation of 95% confidence interval (CI) using log–log transformation were reported. The differences in survival probabilities between the SL and non-SL groups were assessed using the log-rank test. The median follow-up time was calculated using the Schemper and Smith method [15]. Patients’ data were last updated on August 01, 2017. Patients, who were either alive on that date or lost to follow-up, were censored in survival analysis.

The identification of factors that independently predicted receiving second-line therapy was conducted using a two-step procedure based on binominal logistic regression. In the first step, all factors were included in univariable analysis and these factors that reached *P* value less than 0.1 were included in the second step, i.e. multivariable analysis based on step-wise forward selection with significance level of 0.05 for entering and removing variables. Factors that remained significant in the second step contributed to the final model. The model performance was assessed with Nagelkerke’s R^2^ and bias-corrected Nagelkerke’s *R*^2^ as global goodness-of-fit measures, the Hosmer–Lemeshow test for calibration, an area under receiver operating characteristic curve (AUC) and bias-corrected AUC for discrimination.

To assess the robustness of the model, internal validation was performed using two bootstrap procedures that generated new datasets by taking samples from original dataset using random sampling with replacement. In the first procedure, 1000 new datasets were generated and binominal logistic regression was repeated for each sample, using variables selected in the final model. The odds ratios (ORs) with new 95% CIs and *P* values were produced and compared to those of the model derived from original dataset. In the second procedure, another 1000 bootstrap datasets entered the same modelling process used to derive the final model from the original dataset. Factors that appeared in more than 50% of computed models were considered to be significant [20, 21].

Cases with variables that contained missing data were excluded from analyses that involved those variables. *P* values less than 0.05 (two-sided) indicated statistical significance for all tests, except univariable logistic regressions where the cut-off level of 0.1 was used. All statistical procedures were performed using Stata, version 14.2 (StataCorp, College Station, Texas, USA) and R, version 3.2.5 (The R Foundation for Statistical Computing, Vienna, Austria) with the rms package, version 5.1-0.

## Results

### Characteristics of the two groups

Overall, 326 patients treated with first-line TT were screened. Two hundred and sixty-seven (267 [100%]) patients had known second-line TT status and, therefore, were included in the analysis. One hundred and fifty-two (152 [57%]) patients had received second-line TT (everolimus − 117/152 [77%], axitinib − 32/152 [21%], and cabozantinib − 3/152 [2%]) and contributed to the SL group. The remaining 115 [43%] patients were not eligible to receive any subsequent systemic treatment and were assigned to the non-SL group. The detailed characteristics collected at the time of first-line TT initiation are presented in Table 1. Patients in the SL-group had less frequent diagnosis-to-treatment interval < 1 year and Fuhrman grade 3–4 than patients in the non-SL group. At the same time, patients receiving second-line TT presented with better performance status and were more frequently assigned to the International Metastatic Database Consortium (IMDC) favourable- and intermediate-risk groups at treatment initiation than patients with no systemic treatment beyond first-line. The SL-group was characterised with lower total number of metastatic sites, and lower proportion of patients had bone, liver and brain metastases. Patients in the SL-group at treatment initiation had not only higher levels of haemoglobin and albumin concentration, but also lower levels of corrected calcium concentration and platelet count. There were no significant differences between the two groups in terms of first-line targeted drug or other characteristics.

**Table 1:** Patients characteristics at the start of first-line TT (total *N* = 267)

Variable	The SL group (*N* = 152)	The non-SL group (*N* = 115)	*P*
Age, years: median (range)	62 (25–83)	61 (22–85)	0.656
Male: *n* (%)	102 (67)	81 (70)	0.562
BMI [kg/m^2^]: median (range)	25.7 (17.1–48.8)^a^	26.0 (16.8–39.6)^b^	0.433
Time since diagnosis to first-line TT initiation < 1 year: *n* (%)	66 (43)	66 (57)	0.024
Karnofsky PS: *n* (%)			< 0.001
100	79 (52)	23 (20)	
80–90	72 (47)	84 (73)	
< 80	1 (< 1)	8 (7)	
Primary tumour site, right: *n* (%)	69 (45)	60 (52)	0.272
Fuhrman grade, 3–4: *n* (%)	49 (35)^c^	54 (52)^d^	0.008
Non-clear cell histology: *n* (%)	10 (7)	6 (5)	0.643
Sarcomatoid features: *n* (%)	8 (5)	7 (6)	0.772
Number of metastatic sites: median (range)			< 0.001
Metastatic sites: *n* (%)			
Lung	112 (74)	86 (75)	0.839
Lymph nodes	71 (47)	65 (57)	0.112
Bone	41 (27)	46 (40)	0.025
Liver	26 (17)	33 (29)	0.024
Pancreas	14 (9)	11 (10)	0.922
Suprarenal gland	21 (14)	26 (23)	0.062
Brain	1 (< 1)	15 (13)	< 0.001
Local recurrence	32 (21)	35 (30)	0.08
Contralateral kidney	13 (9)	7 (6)	0.449
Other soft tissues	30 (20)	38 (33)	0.013
Haemoglobin [g/dl]: median (range)	13.1 (9.6–19.1)	11.8 (8.9–17.4)	< 0.001
Corrected calcium [mg/dl]: median (range)	9.5 (8.0–11.3)	9.6 (6.8–14.7)	0.043
Lactate dehydrogenase [U/l]: median (range)	177 (106–406)^e^	184 (115–1185)^f^	0.285
Albumin [g/dl]: median (range)	4.3 (2.9–5.6)^e^	3.9 (2.3–5.9)	< 0.001
WBC [× 10^3^/ml]: median (range)	7.6 (3.4–15.4)	7.8 (3.5–20.5)	0.28
Neutrophil count [× 10^3^/ml]: median (range)	4.8 (2.0–11.5)	5.1 (2.2–19.1)	0.09
Platelet count [× 10^3^/ml]: median (range)	250 (101–831)	299 (140–966)	< 0.001
Lymphocyte count [× 10^3^/ml]: median (range)	1.6 (0.4–4.56)	1.6 (0.2–4.8)	0.168
IMDC risk group: *n* (%)			< 0.001
Favourable	69 (46)	25 (22)	
Intermediate	75 (49)	66 (57)	
Poor	8 (5)	24 (21)	
Prior immunotherapy: *n* (%)	15 (10)	14 (12)	0.549
First-line TT therapy: *n* (%)			0.075
Sunitinib	114 (75)	83 (72)	
Pazopanib	23 (15)	27 (23)	
Sorafenib	15 (10)	5 (4)	

*BMI* body mass index, *IMDC* International Metastatic Renal Cell Carcinoma Database Consortium, *KPS* Karnofsky performance status, *LDH* lactate dehydrogenase, *LLN* lower limit of normal, *SL* second-line, *TT* targeted therapy, *ULN* upper limit of normal, *WBC* white blood count

^a^Number of evaluated patients: 143

^b^Number of evaluated patients: 96

^c^Number of evaluated patients: 139

^d^Number of evaluated patients: 103

^e^Number of evaluated patients: 148

^f^Number of evaluated patients: 110

### Survival results

**Fig. 1:**
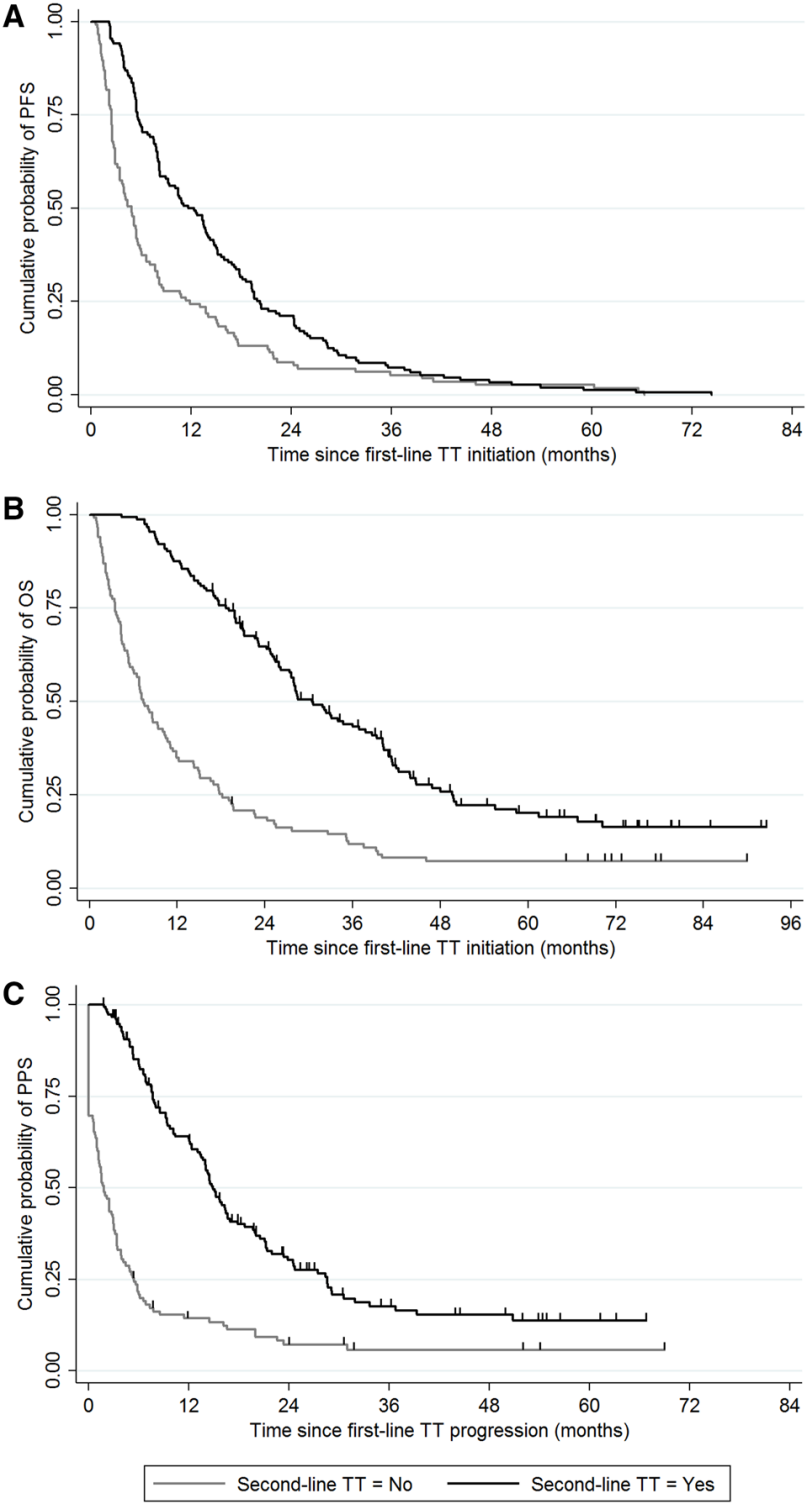
The Kaplan–Meier curves for **a** progression-free survival (PFS), **b** overall survival (OS), and **c** post-progression survival (PPS) stratified by second-line targeted therapy (TT) status

The median follow-up time for the whole cohort of patients was 69.3 months (95% CI 64.1–73.1). The median follow-up time was 69.2 months (95% CI 58.5–74.7) in the SL-group and 71.2 months (95% CI 65.0–78.0) in the non-SL group, respectively. The follow-up time did not differ between the two groups (*P* = 0.496). The median PFS was 8.0 months (95% CI 6.7–9.4), the median OS was 20.0 months (95% CI 17.5–24.7) and the median PPS was 7.7 months (95% CI 6.1–10.2) for all analysed patients. The median PFS was 11.7 months (95% CI 9.0–14.1) and 4.9 months (95% CI 3.5–5.6) for the SL-group and the non-SL group, respectively. The median OS was 30.4 months (95% CI 26.2–37.8) and 7.4 months (95% CI 5.5–10.3) for the SL-group and the non-SL group, respectively. The median PPS was 14.9 months (95% CI 13.5–16.7) and 1.9 months (95% CI 1.2–3.0) for the SL-group and the non-SL group, respectively. The SL-group had significantly longer PFS (*P* < 0.001), OS (*P* < 0.001) and PPS (*P* < 0.001) than the non-SL group (Fig. 1A-C).

### Model building and validation

After performing a series of univariable binominal logistic regressions, 17 factors were found to have an influence on the probability of having second-line TT (Table 2). On the multivariable analysis, five factors remained significant and contributed to the final model. Four of them were associated with decreased probability of having second-line therapy: the presence of brain metastases (OR 0.084, 95% CI 0.010–0.707), number of metastatic sites (OR 0.740, 95% CI 0.575–0.953 per each site), platelet count (OR 0.971, 95% CI 0.947–0.997, per 10^4^/ml) and lactate dehydrogenase (LDH) level (OR 0.952, 95% CI 0.910–0.997 per 10 units/l), while albumin concentration was associated with increased probability (OR 1.924, 95% CI 1.057–3.503 per 1 g/dl). The model showed satisfactory calibration (the Hosmer–Lemeshow test *P* value = 0.133), discrimination (AUC = 0.750, bias-corrected AUC = 0.736) and global fit (Nagelkerke’s *R*^2^ = 0.277, bias-corrected Nagelkerke’s *R*^2^ = 0.231). In the first validation procedure, all five model covariates remained statistically significant after repeating the regression on 1000 bootstrap samples. In the second validation procedure, four factors: number of metastatic sites, LDH, platelet count and albumin concentration appeared in more than a half of 1000 newly constructed models (51, 52, 56 and 52%, respectively), whereas the brain metastases status did not (45%) (Table 3).

**Table 2:** Results of univariable and multivariable binominal logistic regression with second-line targeted therapy status as a dependent variable

Variable	Univariable		Multivariable	
	OR (95% CI)	*P*	OR (95% CI)	*P*
Age	1.00 (0.976–1.025)	0.986		
Gender				
Male	1			
Female	1.168 (0.691–1.973)	0.562		
BMI^a^	1.030 (0.976–1.087)	0.281		
Time since first-line TT initiation				
≥ 1 year	1			
< 1 year	0.570 (0.349–0.929)	0.024		
KPS				
≥ 80%	1			
< 80%	0.089 (0.011–0.719)	0.023		
Primary tumour site				
Right	1			
Left	1.312 (0.807–2.133)	0.273		
Fuhrman grade^b^				
1–2	1			
3–4	0.494 (0.294–0.831)	0.008		
Histology				
Clear-cell	1			
Other	1.279 (0.451–3.628)	0.643		
Sarcomatoid features				
No	1			
Yes	0.857 (0.302–2.436)	0.772		
No. of metastatic sites	0.600 (0.483–0.743)	< 0.001	0.740 (0.575–0.953)	0.020
Lung metastases				
No	1			
Yes	0.944 (0.542–1.643)	0.839		
Lymph nodes metastases				
No	1			
Yes	0.674 (0.414–1.098)	0.113		
Bone metastases				
No	1			
Yes	0.554 (0.330–0.929)	0.025		
Liver metastases				
No	1			
Yes	0.513 (0.286–0.920)	0.025		
Pancreas metastases				
No	1			
Yes	0.959 (0.418–2.199)	0.922		
Suprarenal gland metastases				
No	1			
Yes	0.549 (0.291–1.035)	0.064		
Brain metastases				
No	1			
Yes	0.044 (0.006–0.340)	0.003	0.084 (0.010–0.707)	0.023
Local recurrence				
No	1			
Yes	0.610 (0.349–1.063)	0.081		
Contralateral kidney metastases				
No	1			
Yes	1.443 (0.557–3.741)	0.451		
Other soft tissues metastases				
No	1			
Yes	0.498 (0.285–0.870)	0.014		
Haemoglobin [g/dl]	1.277 (1.114–1.465)	< 0.001		
Corrected calcium [mg/dl]	0.737 (0.520–1.045)	0.087		
Lactate dehydrogenase [× 10 U/l]^c^	0.952 (0.918–0.986)	0.007	0.952 (0.910–0.997)	0.035
Albumin [g/dl]^d^	3.379 (2.049–5.572)	< 0.001	1.924 (1.057–3.503)	0.032
WBC [× 10^3^/ml]	0.919 (0.830–1.017)	0.101		
Neutrophil count [× 10^3^/ml]	0.863 (0.763–0.977)	0.020		
Platelet count [× 10^4^/ml]	0.967 (0.948–0.987)	0.002	0.971 (0.947–0.997)	0.027
Lymphocyte count [× 10^3^/ml]	1.326 (0.953–1.843)	0.094		
Prior immunotherapy				
No	1			
Yes	0.790 (0.365–1.710)	0.549		
First-line TT therapy				
Sunitinib	1			
Pazopanib	2.184 (0.764–6.247)	0.145		
Sorafenib	0.620 (0.332–1.157)	0.133		

*BMI* body mass index, *CI* confidence interval, *KPS* Karnofsky performance status, *LDH* lactate dehydrogenase, *LLN* lower limit of normal, *OR* odds ratio, *SL* second-line, *TT* targeted therapy, *ULN* upper limit of normal, *WBC* white blood count

^a^Number of evaluated patients: 239

^b^Number of evaluated patients: 242

^c^Number of evaluated patients: 258

^d^Number of evaluated patients: 263

**Table 3:** The results of bootstrap procedures for multivariable binominal logistic regression with second-line targeted therapy status as dependent variable

Variable	Frequency of Entry (%)	OR (95% CI)	*P*
Brain metastases	45	0.084 (0.026–0.274)	< 0.001
No. of metastatic sites	51	0.740 (0.568–0.965)	0.026
Platelet count [× 10^4^/ml]	56	0.997 (0.994–0.999)	0.042
Lactate dehydrogenase [× 10 U/l]	52	0.995 (0.991–0.999)	0.020
Albumin [g/dl]	52	1.924 (1.014–3.650)	0.045

*CI* confidence interval, *OR* odds ratio

The regression equation was used to construct a calculator, named MRCCSECLINE, which gives the probability of having second-line TT in MRCC patients. A free version of the calculator is available at http://www.r-calc.com.

## Discussion

Between 2006 and 2011, the use of TTs in patients with mRCC increased from below 23% to over 70% [9]. The population-wide studies show that currently, approximately 95% of all patients are treated with TT of at least one line ^8^. The best clinical outcomes are achieved with sequential use of targeted drugs which is a mainstream in present and near-future therapy of mRCC [22]. However, about 50% of patients will not receive second-line treatment and, therefore, their survival benefit will strictly depend on first-line treatment efficacy. Thus, the proper identification of patients ineligible for subsequent therapy becomes essential in a construction of a long-term treatment plan. Herein, we aimed to develop a calculator that could predict the probability of second-line treatment based on patient characteristics present at first-line therapy initiation.

In our study, the proportion of patients not receiving second-line therapy was 43% and was similar to those reported previously [6–10]. Patients in the SL and non-SL groups differed in numerous baseline features, including performance status, diagnosis-to-treatment interval, number of metastatic sites, presence of bone, liver and brain metastases, haemoglobin, calcium, albumin and platelet count, which are widely recognised as independent RCC prognostic factors [23]. Not surprisingly, it translated into more frequent assignment of patients in the non-SL group to the IMDC intermediate- and poor-risk groups than those in the SL group. Additionally, patients in the SL group had less frequently Fuhrman grade 3–4 histopathology which stays in accordance with previous report stating that patients with grade 1 tumour received second-line therapy more frequently than those with grade 2/3 tumours [6]. Likewise in other reports, in patients ineligible for second-line treatment—the IMDC status at first-line treatment initiation is more often intermediate (~ 50) or poor (~ 40%), age is higher (age > 75 in ~ 40%), nephrectomy was less often performed (~ 60%), but metastases are more often found in liver (~ 20%), bones (~ 30%), skin/soft-tissue (~ 30%) and central nervous system (13%) [12].

Overall, five factors were recognised as independently influencing the probability of receiving second-line treatment: platelet count, LDH and albumin levels, total number of metastatic sites and the presence of brain metastases. Within these, brain metastases status had the largest impact on the calculated probability. For example, a hypothetical patient with platelet count of 200 000/ml, LDH level of 100 U/I, albumin concentration of 4 g/dl and metastases to two organs other than brain has the probability of 80% to receive second-line therapy, but only 25% if brain is within the two organs affected by the metastatic process. Notably, the choice of first-line agent may not be predictive for receiving second-line treatment as it was reported previously by Leavy et al. [10].

The internal validation confirmed the appropriate construction of our model because all variables of the regression formula in the first bootstrap procedure and four (platelet count, LDH and albumin levels, total number of metastatic sites) in the second bootstrap procedure remained significant. The brain metastases status did not reach the planned 50% frequency of entry probably due to statistical uncertainty caused by small proportion of patients who had brain metastases (6% in the analysed cohort).

The median follow-up time in our study was about was almost six years which is one of the longest reported in the literature [24, 25]. Such long follow-up increases the reliability of the research results because our study captures more patients who might not be recognised as receiving second-line in a case of long duration of first-line treatment and short follow-up period. The median OS of 30.4 months for the SL-group is very close to 29.5 months reported recently for sunitinib-everolimus sequential treatment which actually was a common therapeutic strategy in our patients [26]. The median OS of 7.4 months in the non-SL group echoes the median OS of patients assigned to the IMDC poor-risk group in other populations studies [23]. Interestingly, PFS and PPS were also shorter in patients not receiving second-line therapy, which may support the thesis that first-line PFS may act as a surrogate end-point for overall OS [27]. What is more, Eggers et al. reported that early progression, defined as progression within 6 months since the start of first-line therapy, was associated with lower probability of having second-line treatment [6]. However, this parameter will not be known at the start of first-line treatment.

Nowadays, everolimus, axitinib, nivolumab and cabozantinib are used extensively in patients who progressed on prior antiangiogenic TKI therapy. Currently with multiple treatment options, including immunotherapy, reimbursed in selected countries, optimal choice and sequencing is more and more challenging [28]. The Bayesian fixed-effects network meta-analysis model comparing PFS and OS of cabozantinib versus everolimus, nivolumab, axitinib, sorafenib and best supportive care (BSC) showed that cabozantinib was superior to all its comparators with a higher probability of longer PFS and OS during 3 years, but in the Gompertz model nivolumab was preferred after 24 months [29]. These trials are expected to determine the shift of everolimus to the third-line and subsequent lines of treatment if positive in future in selected countries with more robust resources allocated to healthcare system. Unfortunately, it is very unlikely that prospective trials comparing head to head the activity of axitinib, cabozantinib, lenvatinib and nivolumab will be conducted. At this point of time, clinicians still lack biomarkers and recommendation on the optimal sequence of treatment in individual cases. We believe that selected clinical variables can help physicians to make decisions in the future [30] and personalised decisions could be supported with calculator developed within this project.

The limitations of the study include its retrospective design and lack of external validation in another cancer centre. Nevertheless, the proposed model was successfully validated using two internal bootstrap procedures and has shown good statistical performance. Similar models, including nomograms and calculators are being developed in the field of medical oncology practice including advanced oesophagogastric adenocarcinoma nomogram for patients undergoing first-line combination chemotherapy [31], advanced urothelial carcinoma patients to estimate the activity of second-line therapy [32] or advanced luminal subtype breast cancer patients to estimate PFS after first-line therapy [33]. Medical calculators incorporating prognostic factors may facilitate the evaluation of outcomes across different groups of patients before treatment enrolment. We believe that the MRSCCSECLINE calculator should contribute to informed, evidence-based clinical decision making and optimise medical practice as well as future trial recruitment and design.

## Conclusions

The MRCCSECLINE calculator developed in our study may be a useful tool for clinicians to identify those mRCC patients, who are unlikely to receive second-line treatment, and subsequently, to help determine the most optimal, long-term treatment plan at the beginning of systemic TT. However, independent validation of the calculator in prospective trials and additional studies to identify other tumour-specific prognostic factors for all therapies are needed in the future. Medical calculator potentially facilitates evidence-based treatment decisions, individualised risk assessment and helps to select suitable agents or BSC for second-line treatment of mRCC.

Until today, the MSKCC score and first-line treatment type were considered as predictive factors of receiving second-line therapy. No second-line treatment-oriented nomograms or prediction scales are available. We have evaluated 17 clinical and biochemical parameters that are widely evaluated at RCC first line treatment initiation and defined these that impact first-line treatment survival and, therefore, second line treatment enrolment. The presence of brain metastases, number of metastatic sites abnormal platelet count and lactate dehydrogenase level are found in patents that are at a risk of nor eligibility of second line treatment. Normal albumin concentration is associated with increased probability or sequential treatment. Based on identified factors, multi-factorial model and on-line calculator was built for treatment prediction. The MRCCSECLINE calculator may become a practical tool to identify mRCC patients, who are unlikely to receive second-line treatment, and, therefore, to help determine optimal first lime treatment to obtain best response and treatment safety.
